# Protocol for a systematic review of prognosis after mild traumatic brain injury: an update of the WHO Collaborating Centre Task Force findings

**DOI:** 10.1186/2046-4053-1-17

**Published:** 2012-02-23

**Authors:** Carol Cancelliere, J David Cassidy, Pierre Côté, Cesar A Hincapié, Jan Hartvigsen, Linda J Carroll, Connie Marras, Eleanor Boyle, Vicki Kristman, Ryan Hung, Britt-Marie Stålnacke, Peter Rumney, Victor Coronado, Lena W Holm, Jörgen Borg, Catharina Nygren-de Boussard, Jean-Luc af Geijerstam, Michelle Keightley

**Affiliations:** 1 Division of Health Care and Outcomes Research, Toronto Western Research Institute, University Health Network, Ontario, Canada; 2 Institute of Sport Science and Clinical Biomechanics, Faculty of Health, University of Southern Denmark, Odense, Denmark; 3 Division of Epidemiology, Dalla Lana School of Public Health, University of Toronto, Ontario, Canada; 4 Institute of Health Policy, Management and Evaluation, University of Toronto, Ontario, Canada; 5 Nordic Institute of Chiropractic and Clinical Biomechanics, Odense, Denmark; 6 Department of Epidemiology, School of Public Health, University of Alberta, Alberta, Canada; 7 Alberta Centre for Injury Control and Research, Alberta, Canada; 8 Department of Neurology, University of Toronto, Ontario, Canada; 9 Movement Disorders Clinic, Toronto Western Hospital, Ontario, Canada; 10 Department of Biostatistics, Dalla Lana School of Public Health, University of Toronto, Ontario, Canada; 11 Department of Health Sciences, Lakehead University, Ontario, Canada; 12 Institute for Work and Health, Toronto, Ontario, Canada; 13 Department of Rehabilitation and Complex Continuing Care, Holland Bloorview Kids Rehabilitation Hospital, Toronto, Ontario, Canada; 14 Department of Pediatrics, University of Toronto, Ontario, Canada; 15 Department of Community Medicine and Rehabilitation, Umeå University, Sweden; 16 National Center for Injury Prevention and Control, Centers for Disease Control and Prevention, Atlanta, USA; 17 Institute of Environmental Medicine, Karolinska Institutet, Stockholm, Sweden; 18 Department of Rehabilitation Medicine, Danderyd University Hospital, Karolinska Institutet, Stockholm, Sweden; 19 Department of Medicine, Unit of Clinical Epidemiology, Karolinska Institutet, Stockholm, Sweden; 20 Department of Occupational Science and Occupational Therapy, University of Toronto, Ontario, Canada

## Abstract

**Background:**

Mild traumatic brain injury (MTBI) is a major public-health concern and represents 70-90% of all treated traumatic brain injuries. The last best-evidence synthesis, conducted by the WHO Collaborating Centre for Neurotrauma, Prevention, Management and Rehabilitation in 2002, found few quality studies on prognosis. The objective of this review is to update these findings. Specifically, we aim to describe the course, identify modifiable prognostic factors, determine long-term sequelae, and identify effects of interventions for MTBI. Finally, we will identify gaps in the literature, and make recommendations for future research.

**Methods:**

The databases MEDLINE, PsychINFO, Embase, CINAHL and SPORTDiscus were systematically searched (2001 to date). The search terms included 'traumatic brain injury', 'craniocerebral trauma', 'prognosis', and 'recovery of function'. Reference lists of eligible papers were also searched. Studies were screened according to pre-defined inclusion and exclusion criteria. Inclusion criteria included original, published peer-reviewed research reports in English, French, Swedish, Norwegian, Danish and Spanish, and human participants of all ages with an accepted definition of MTBI. Exclusion criteria included publication types other than systematic reviews, meta-analyses, randomized controlled trials, cohort studies, and case-control studies; as well as cadaveric, biomechanical, and laboratory studies. All eligible papers were critically appraised using a modification of the Scottish Intercollegiate Guidelines Network (SIGN) criteria. Two reviewers performed independent, in-depth reviews of each eligible study, and a third reviewer was consulted for disagreements. Data from accepted papers were extracted into evidence tables, and the evidence was synthesized according to the modified SIGN criteria.

**Conclusion:**

The results of this study form the basis for a better understanding of recovery after MTBI, and will allow development of prediction tools and recommendation of interventions, as well as informing health policy and setting a future research agenda.

## Background

Traumatic brain injury (TBI) is a leading cause of death and disability [1]. Increasingly, mild (M)TBI (concussion) has been recognized as a public-health concern especially for teenagers and young adults [2] because it can potentially lead to significant disruptions in education and working life [3]. It is estimated that MTBI represents 70% to 90% of all treated cases of TBI, and that the incidence of hospital treatment in adults with MTBI ranges from about 100 to 300 per 100,000 person-years [2]. However, because a large number of MTBI cases are not treated in hospital, the incidence of all MTBI among adults is likely to be in excess of 600 per 100,000 person-years [2]. The economic effect of MTBI is substantial, accounting for approximately 44% of the US$60 billion annual cost of TBI in the USA [4,5]. It has been reported that most of these cases recover within 3 to 12 months; however, some studies suggest that a considerable minority continue to report distressing symptoms that persist [6]. Patients often experience a combination of physical, emotional, and cognitive symptoms, collectively known as post-concussion syndrome (PCS) [6]. Commonly reported PCS symptoms include headaches, balance problems, dizziness, fatigue, depression, anxiety, irritability, and memory and attention difficulties, which can have a considerable negative effect on the patient's ability to return to pre-injury function, work, and/or school [6]. In addition, there is some evidence that MTBI is associated with an increased risk for certain neurological and/or psychiatric disorders, including Parkinson's disease (PD), early-onset dementia, chronic traumatic encephalopathy, and schizophrenia [7-11].

In its last review, the WHO Collaborating Centre for Neurotrauma, Prevention, Management and Rehabilitation Task Force found that studies addressing issues of prognosis had a low scientific quality [12]. Specifically, the Task Force found a scarcity of good-quality studies on prognostic factors in both older people and children, and very few scientifically admissible studies on the health effects of multiple concussions [12]. There were too few studies on the long-term consequences of MTBI following repeated head injuries, such as in hockey and/or American football, to make any strong conclusions [12]. Additionally, the previous review identified only sparse evidence on interventions after MTBI [13]. The presence of head injuries in military personnel is also an important concern, given the prevalence of blast-related war injuries sustained in areas such as Iraq and Afghanistan [14]. In addition to these knowledge gaps, the Task Force did not find any acceptable studies on return to work or school after MTBI, nor did it find any studies that had developed prediction rules to identify those at risk for not recovering and/or developing longer-term health problems [12].

Prognosis is a central issue in health care, which is related to both the identification of individuals at risk for poor recovery, and identification of modifiable risk factors and feasible treatment strategies [15]. From a diagnostic point of view, screening for those at risk for poor recovery could potentially help to triage patients into more effective health interventions. Additionally, any intervention can also be viewed as a modifiable prognostic factor, and to be a useful intervention, it must improve prognosis. Given that resources are limited, it is important to compare the effectiveness of interventions versus the modification of prognostic factors (for example, psychosocial factors) in reducing the burden of disease in both patients and society at large. In order to develop prediction rules to aid clinical decision-making, it is of the utmost importance that information on prognostic factors is available from scientifically valid studies. Given the numbers of people affected by MTBI, the ability to triage patients at risk for poor recovery to allow focused and cost-effective management strategies becomes an important health policy issue.

In this study, we proposed to update the WHO Collaborating Centre Task Force findings on prognosis in both the adult and pediatric populations to: 1) describe the course and identify prognostic factors of recovery after MTBI, for example, following work injuries, traffic injuries, sports injuries and military-related MTBI; 2) describe the long-term sequelae of MTBI (such as brain tumor, dementia, PD, Alzheimer's disease, chronic pain, chronic traumatic encephalopathy, attention deficit and hyperactivity disorder, and disability); 3) identify modifiable prognostic factors; 4) identify candidate prognostic factors to be used to develop clinical prediction rules for early identification of patients at risk for poor recovery after MTBI; 5) make clinical and methodological recommendations for future research to address prognosis after MTBI; and 6) evaluate the effectiveness of clinical interventions for the management of MTBI (for example, medical, rehabilitative, and/or vocational).

## Methods

This project was funded by the Ontario Neurotrauma Foundation (Grant Ref: 2010-ABI-MTBIWHO-871). The funder was not involved in the design or preparation of the study protocol; in the management of the project, analysis or interpretation of data; or in the preparation of the final report and publications.

We conducted a systematic review and best-evidence synthesis in order to update the findings of the WHO Collaborating Task Force. The review was conducted and reported in compliance with the Preferred Reporting Items for Systematic Reviews and Meta-Analyses (PRISMA) guidelines [16]. In accordance with the guidelines, our systematic review protocol was registered with the International Prospective Register of Systematic Reviews (PROSPERO) [17] on 11 July 2011 and was last updated on 19 January, 2012 (registration number CRD42011001410).

### Literature search

The electronic databases MEDLINE, PsychINFO, Embase, CINAHL and SPORTDiscus were systematically searched from 1 January 2001 to 30 June 2011. This date was chosen because the last best-evidence synthesis conducted by the WHO Collaborating Centre Task Force, included a literature search to the end of 2000.

The search strategies were designed and tested for sensitivity with the assistance of an information scientist (see Additional file 1). In the future, the searches will be updated to capture the most recent literature. The reference lists of papers meeting the eligibility criteria were screened for additional potentially relevant papers that may have been missed by our electronic searches. Additionally, Task Force members provided information about studies of which they had knowledge but which were not found in the databases or reference lists.

### Eligibility criteria

Titles and abstracts were screened for eligibility according to the following inclusion and exclusion criteria.

#### Inclusion criteria

• Languages: English, French, Swedish, Norwegian, Danish and Spanish.

• Publication type: original research manuscripts published in peer-reviewed journals.

• Study design: systematic reviews, meta-analyses, randomized controlled trials (RCTs), case-control studies, and cohort studies. All included studies had to include a minimum of 30 MTBI cases, so that a better level of precision and confidence in the results could be achieved.

• Study population: human participants of all ages. Participants could consist of a mixed group of TBI severity (mild, moderate or severe) only if the results were stratified by severity and the MTBI subjects could be clearly identified.

• Case definition: Studies had to state a clear case definition for MTBI that falls within the definitions provided by the WHO Collaborating Centre Task Force and the Centers for Disease Control and Prevention (CDC). The Task Force states that 'MTBI is an acute brain injury resulting from mechanical energy to the head from external physical forces. Operational criteria for clinical identification include: (i) one or more of the following: confusion or disorientation, loss of consciousness for 30 minutes or less, post-traumatic amnesia for less than 24 hours, and/or other transient neurological abnormalities such as focal signs, seizure, and intracranial lesion not requiring surgery; and (ii) Glasgow Coma Scale score of 13-15 after 30 minutes post-injury or later upon presentation for healthcare. These manifestations of MTBI must not be due to drugs, alcohol, medications, caused by other injuries or treatment for other injuries (e.g. systemic injuries, facial injuries or intubation), caused by other problems (e.g. psychological trauma, language barrier or coexisting medical conditions) or caused by penetrating craniocerebral injury' [18]. People with fractured skulls could be included if they met this case definition.

The CDC provided an additional definition based on clinical records data. Using this definition, a person is considered to have an MTBI if they have a documented Abbreviated Injury Severity Scale score of 2 for the head region [1]. An administrative data definition for surveillance or research was also provided [1]. Specifically, cases of MTBI were recognized if the patients were assigned certain diagnostic codes on the *International Classification of Diseases, Ninth Revision, Clinical Modification *(ICD-9-CM) (Table 1).

**Table 1:** *International Classification of Diseases, Ninth Revision, Clinical Modification *(ICD-9-CM) codes for mild traumatic brain injury

ICD-9-CM first four digits	**ICD-9-CM fifth digit**^a^
800.0, 800.5, 801.0, 801.5, 803.0, 803.5, 804.0, 804.5, 850.0, 850.1, 850.5 or 850.9	0, 1, 2, 6, 9, or missing

854.0	1, 2, 6, 9, or missing

959.0^b^	1

^a^Sometimes a fifth digit is provided in the code (for example, ICD-9-CM 800.00).

^b^The current inclusion of code 959.01 is provisional.

• Study outcomes: self-rated recovery, functional recovery (for example, return to activities, work or school), improvement in clinical outcomes such as memory and concentration, and risk for long-term sequelae of MTBI (for example, dementia and PD, among others).

• Examination of modifiable prognostic factors and clinical prediction rules for diagnosis or triage of patients with MTBI.

#### Exclusion criteria

• Publication type: narrative reviews, letters, editorials, commentaries, unpublished manuscripts, dissertations, government reports, books and book chapters, conference proceedings, meeting abstracts, lectures and addresses, and consensus development statements (including guideline statements).

• Study design: cross-sectional studies, case reports and series, qualitative studies, nonsystematic reviews, studies that did not report methods, and cadaveric, biomechanical, and laboratory studies.

• Study population: animals.

• Case definition: neck fractures and open or penetrating head injury, non-traumatic brain injury, or MTBI caused by violence/assault in the civilian population or child abuse (for example, shaken baby syndrome). Cases of MTBI sustained by military personnel during combat or other military-associated operations could be included provided that they met the MTBI case definition stated.

#### Screening

For the first level of screening, one reviewer read the titles of all the citations retrieved from the electronic database searches and removed all citations that were clearly not related to TBI. The second level of screening involved abstract review. Full-text articles were obtained for all abstracts except for those that clearly did not meet the eligibility criteria. If after analyzing the full text, the eligibility of an article was still uncertain, a second reviewer undertook a full-text analysis of the article to determine eligibility. A third reviewer was consulted in the event of any disagreements. Level of agreement on study eligibility was tested using the kappa statistic and 95% confidence interval.

#### Critical appraisal of the literature

All eligible papers will be independently reviewed by two reviewers using modified Scottish Intercollegiate Guidelines Network (SIGN) criteria. The reviewers were international scientists and experts in MTBI management and research, and/or had experience in systematic review methodology. The purpose of the SIGN criteria was to assess the internal validity of each study [19,20].

We modified the SIGN checklists by adding a few supplementary items (see Additional file 2). Given that there were various definitions of MTBI in the literature, we added an item to the checklists to assess whether or not a clear case definition had been provided. We added a section to describe the main strengths and weaknesses of the study, and a general 'Comments' section allowing for further sentiments to be expressed that could facilitate the synthesis of our final reports. Because our review focused on prognosis as well as interventions, some of the wording had to be modified from the original SIGN forms. Some of the original questions were divided into two separate questions: for example, '...the method of outcome assessment is valid and reliable' was changed to (i) '...the method of outcome assessment is reliable,' and (ii) '...the method of outcome assessment is valid,' because it was possible for a method to be reliable without being valid. We also added a section about which references need to be checked, as our method of ascertaining articles involved scanning the reference lists of all eligible articles. Finally, we omitted the 'description of the study' section because data extraction was to be performed at a separate time. However, the information to satisfy the items in this section was accounted for and made available in our evidence tables, such as inclusion/exclusion criteria, follow-up periods, and patient characteristics.

Standardized instructions have been provided to assist reviewers with their assessments (see Additional file 3). A consensus method was used to solve disagreements about risk for bias assessment, and a third reviewer was consulted if disagreements persisted. Level of agreement on study admissibility was tested using the kappa statistic with its 95% confidence interval.

#### Data extraction

Data from admissible papers (that is, those with a low risk for bias) was extracted by two independent reviewers. After consensus had been reached, the data were entered into evidence tables, and a third reviewer was consulted if there was disagreement. The data to be extracted were 1) study name, authors and publication date; 2) publication language; 3) publication type; 4) geographic origin; 5) MTBI case definition; 6) study design; 7) study phase according to Côté and colleagues [21]; 8) participant characteristics; 9) prognostic factors/outcomes; 10) follow-up periods; and 11) key findings.

#### Analysis

The data entered into the evidence tables were synthesized according to modified SIGN criteria (see Additional file 4) and used to draw inferences about prognosis and other aims of our review [20]. The wording of these criteria was modified to reflect prognostic studies in addition to intervention studies, and to include any population of interest (not just the Scottish population). A meta-analysis was carried out if the study populations and methods were sufficiently comparable across studies to allow for pooling of the results. A best-evidence synthesis [22] was performed to provide clear and useful conclusions based on the best available evidence. This type of synthesis incorporates explicit *a priori *systematic literature-search methods. It also provides a detailed analysis of the critical review process, study selection, and study characteristics in order to justify the review findings.

We also synthesized the evidence using the phases of study framework described by Côté and colleagues [21] which is a modification of the work of Altman and Layman [23]. Phase 1 studies were hypothesis-generating investigations that explored the associations between potential prognostic factors and disease outcomes in a descriptive or univariate way. Phase II studies were extensive exploratory analyses that focused on particular sets of prognostic factors, or attempted to discover which factors have the highest prognostic value. Lastly, phase III studies were large confirmatory studies of explicit pre-stated hypotheses that allow for a focused examination of the strength, direction, and independence of the proposed relationship between a prognostic factor and the outcome of interest.

Furthermore, our findings were synthesized separately for the adult and pediatric populations, and for specialized populations such as the military and athletes. Specific topics included: 1) clinical course, natural history and prognostic factors for adult and pediatric MTBI; 2) return to work after MTBI; 3) prognosis after sport-related MTBI and return to play; 4) prognosis after MTBI injury during military service; 5) long-term psychosocial issues in adults and children after MTBI; 6) risk for PD after MTBI; 7) risk for dementia after MTBI; and 8) interventions for MTBI in adults and children. Finally, we collected data on the risks of bias for each admissible study (such as confounding, data collection, and outcome assessment), results of the evidence synthesis, and recommendations for future research and clinical practice.

## Discussion

We believe that this update of the WHO Task Force findings on prognosis after MTBI will significantly improve clinical and methodological understanding of recovery after MTBI and its associated factors. First, the investigative team included international experts on clinical and methodological issues in this field. The search strategies that were developed are intentionally sensitive, rather than specific (see Additional file 1). For example, search terms were not restricted to 'mild' or 'minor' brain injury because testing of the searches showed that doing so excluded some relevant articles. In particular, many studies address all levels of TBI and stratify some results by severity. Some of these would have been missed if we had restricted our searches to mild or minor TBI. Publications in French, Swedish, Norwegian, Danish, and Spanish in addition to English are included because some of the reviewers were fluent in one or more of these languages.

We modified the SIGN checklists in order to make our critical review process more relevant to the objectives of our review. Specifically, we made modifications to more clearly assess the presence of biases specific to prognostic studies as opposed to intervention studies, which are the main focus of the SIGN criteria.

We anticipated that the quantity and quality of research on prognosis after MTBI has increased substantively over the past decade for a couple of reasons. First, there is an increasing amount of media attention surrounding the long-term sequelae of MTBI in the athletic and military populations. Secondly, recommendations for improving the quality of research were provided by the Task Force in its last review a decade ago, and we believed that authors would have responded to this with research that was better designed and implemented.

The results of this study should form the basis to better understand recovery after MTBI, to develop prediction tools, inform health policy and clinical management, and to set a future research agenda on prognosis of MTBI. In particular, information on prognostic factors from accepted studies can be used to design and test clinical prediction tools, which could then be used to triage patients into low and high-risk categories for developing on-going symptoms. This in turn can inform early intervention and management. Additionally, this information can be used to target resources towards the modification of appropriate prognostic factors to achieve the best outcomes for patients with MTBI.

## Competing interests

The authors declare that they have no competing interests.

## Authors' contributions

CC coordinated the study, developed the search strategies, registered the protocol and drafted the manuscript. JDC conceived the study, designed it, obtained the funding, and helped to coordinate and draft the manuscript. PC helped to inform the study design and revised the manuscript substantially. All others helped to inform the study design and revise the manuscript. All authors read and approved the final manuscript.

## Supplementary Material

Additional file 1 **Search strategy**. This file provides the list of search terms used to search Medline. Click here for file

Additional file 2 **Risk for bias assessment checklists**. This file contains modified SIGN checklists used to assess the risk for bias of eligible studies. Click here for file

Additional file 3 **Scottish Intercollegiate Guidelines Network (SIGN) notes**. This file contains modified SIGN notes used to aid reviewers in completing the SIGN checklists. Click here for file

Additional file 4 **Scottish Intercollegiate Guidelines Network (SIGN) considered judgment form**. This file contains modified SIGN criteria and notes that were used to synthesize the evidence and make recommendations. Click here for file
