# Supplementary material for: Protocol for a systematic review of prognosis after mild traumatic brain injury: an update of the WHO Collaborating Centre Task Force findings
Source: Syst Rev. 2012 Feb 23;1:17. doi: 10.1186/2046-4053-1-17 (PMC3351713; doi:10.1186/2046-4053-1-17)
Supplement: Additional file 1 — Search strategy. This file provides the list of search terms used to search Medline. [file 2046-4053-1-17-S1.PDF]

Medline Search Strategy

1. exp Brain Edema/
2. exp Cerebrovascular Trauma/
3. exp Craniocerebral Trauma/
4. exp Coma/
5. exp Glasgow Coma Scale/
6. exp Glasgow Outcome Scale/
7. ((brain* or capitis or cerebr* or crani* or hemispher* or inter-crani* or intra-crani* or skull*) adj4 (contusion* or damag* or fractur* or injur* or trauma* or wound*)).ab,ti.
8. ((brain or crani* or cerebr* or head or inter-cran* or intra-cran*) adj4 (bleed* or haematoma* or haemorrhag* or hematoma* or hemorrhag* or pressure)).ti,ab.
9. (Glasgow adj (coma or outcome) adj (scale* or score*)).ab,ti.
10. 'Rancho Los Amigos Scale'.ti,ab.
11. diffuse axonal injur*.ti,ab.
12. ((brain or cerebral or intracranial) adj3 (edema or oedema or swell*)).ab,ti.
13. ((coma* or concuss* or unconscious* or 'persistent vegetative state') adj2 (damag* or fractur* or injur* or trauma* or wound*)).ti,ab.
14. (mtbi or "mild trauma* injur*").tw. or "minor trauma* injur*".mp. [mp=protocol supplementary concept, rare disease supplementary concept, title, original title, abstract, name of substance word, subject heading word, unique identifier]
15. or/1-14
16. exp cohort studies/
17. exp prognosis/
18. exp morbidity/
19. exp mortality/
20. exp survival analysis/
21. exp models, statistical/
22. prognos*.tw.
23. predict*.tw.
24. course*.tw.
25. diagnosed.tw.
26. cohort*.tw.
27. death.tw.
28. or/16-27
29. diagnosed.tw.

30. cohort:.mp.

31. (predictor: or death).tw.

32. exp Models, Statistical/

33. Prognosis/

34. or/29-33

35. 28 or 34

36. 15 and 35

37. exp Rehabilitation, Vocational/

38. exp Employment/

39. exp Work/

40. Sick Leave/

41. Absenteeism/

42. exp Occupational Health/

43. exp Occupational Medicine/

44. exp Disabled Persons/

45. "Recovery of Function"/

46. exp Human Activities/

47. exp Self Care/

48. activities of daily living.tw.

49. (dressing or feeding or eating or toilet$ or bathing or mobil$ or driving or public transport$).tw.

50. ((daily or domestic or house or home) adj5 (activit$ or task$ or skill$ or chore$)).tw.

51. ("work status" or "work capacity").tw.

52. (unemployment or re-employment or underemployment or "job retention").ti,ab.

53. (return* adj2 school).tw.

54. or/37-53

55. 15 and 54

56. exp Dementia/

57. Delirium/ or exp Delirium, Dementia, Amnestic, Cognitive Disorders/

58. dement*.mp. or alzheimer*.tw. [mp=protocol supplementary concept, rare disease supplementary concept, title, original title, abstract, name of substance word, subject heading word, unique identifier]

59. exp Parkinsonian Disorders/

60. parkinson*.tw.

61. or/56-60

62. 15 and 61

63. 36 or 55 or 62

64. animals/ not (humans/ and animals/)

65. 63 not 64
66. limit 65 to (danish or english or french or norwegian or spanish or swedish)
67. limit 66 to yr="2001 -Current"
68. exp "Outcome Assessment (Health Care)"/
69. (intervention* adj3 stud*).tw.
70. 68 or 69
71. 15 and 70
72. 71 not 64
73. limit 72 to (yr="2001 -Current" and (danish or english or french or norwegian or swedish))
74. 67 or 73
75. randomized controlled trial.pt.
76. Randomized controlled trial/
77. Randomized Controlled Trials as Topic/
78. clinical trial.pt.
79. Double-Blind Method/
80. "double blind:".mp.
81. Placebos/
82. placebo:.mp.
83. random:.mp.
84. or/75-83
85. 15 and 84
86. review/
87. (medline or medlars or pubmed or grateful med or CINAHL or scisearch or psychinfo or psycinfo or psychlit or psyclit or handsearch* or hand search* or manual* search* or electronic database* or bibliographic database* or embase or lilacs or scopus or web of science).mp.
88. 86 and 87
89. meta-analysis.mp.
90. meta-analysis as topic/
91. meta-analysis/
92. systematic review*.tw.
93. cochrane database*.jn.
94. or/88-93
95. 15 and 94
96. exp Brain Neoplasms/

97. (cancer* or neoplasm* or tumor* or malign*).mp. and brain.tw. [mp=protocol supplementary concept, rare disease supplementary concept, title, original title, abstract, name of substance word, subject heading word, unique identifier]

98. exp Glioma/

99. 96 or 97 or 98

100. 15 and 99

101. exp Pain/

102. exp Chronic Disease/

103. 101 and 102

104. (chronic* adj3 pain*).mp. [mp=protocol supplementary concept, rare disease supplementary concept, title, original title, abstract, name of substance word, subject heading word, unique identifier]

105. 103 or 104

106. 15 and 105

107. exp Sports/

108. exp Recreation/

109. (return* adj3 play*).tw.

110. 107 or 108 or 109

111. 15 and 110

112. exp Mental Disorders/

113. 15 and 112

114. exp Disability Evaluation/

115. exp "Outcome Assessment (Health Care)"/

116. disab:.tw.

117. 114 or 115 or 116

118. 15 and 117

119. 74 or 85 or 95 or 100 or 106 or 111 or 113 or 118

120. limit 119 to (english language and yr="2001 -Current" and (danish or english or french or norwegian or swedish))

121. animals/ not (humans/ and animals/)

122. 120 not 121
