# Supplementary material for: Protocol for a systematic review of prognosis after mild traumatic brain injury: an update of the WHO Collaborating Centre Task Force findings
Source: Syst Rev. 2012 Feb 23;1:17. doi: 10.1186/2046-4053-1-17 (PMC3351713; doi:10.1186/2046-4053-1-17)
Supplement: Additional file 2 — Risk for bias assessment checklists. This file contains modified SIGN checklists used to assess the risk for bias of eligible studies. [file 2046-4053-1-17-S2.PDF]

# Methodology Checklist 2: Randomized Controlled Trials

SIGN

## SECTION 1: STUDY IDENTIFICATION

| ID: | First author: | Year: | Journal: |
|---|---|---|---|
| Title: | | | |
| Objectives: | | | |
| Language: | | | |
| Country: | Admissible Inadmissible | | Checklist completed by: |

## SECTION 2: INTERNAL VALIDITY

| | *In a well conducted RCT study…* | *In this study this criterion is:* | |
|---|---|---|---|
| 2.1 | The study addresses an appropriate and clearly focused question.<br>Comments: | Well covered<br>Adequately addressed<br>Poorly addressed | Not addressed<br>Not reported<br>Not applicable |
| 2.2 | The definition of MTBI is clear.<br>Comments: | Well covered<br>Adequately addressed<br>Poorly addressed | Not addressed<br>Not reported<br>Not applicable |
| 2.3 | The assignment of subjects to treatment groups is randomized.<br>Comments: | Well covered<br>Adequately addressed<br>Poorly addressed | Not addressed<br>Not reported<br>Not applicable |
| 2.4 | An adequate concealment method is used.<br>Comments: | Well covered<br>Adequately addressed<br>Poorly addressed | Not addressed<br>Not reported<br>Not applicable |
| 2.5 | Subjects and investigators are kept 'blind' to treatment allocation.<br>Comments: | Well covered<br>Adequately addressed<br>Poorly addressed | Not addressed<br>Not reported<br>Not applicable |
| 2.6 | The treatment and control groups are similar at the | Well covered | Not addressed |

| | | |
|---|---|---|
| | start of the trial.<br>Comments: | Adequately addressed / Not reported<br>Poorly addressed / Not applicable |
| 2.7 | The only difference between groups is the treatment under investigation.<br>Comments: | Well covered / Not addressed<br>Adequately addressed / Not reported<br>Poorly addressed / Not applicable |
| 2.8 | All relevant outcomes are measured in a reliable way.<br>Comments: | Well covered / Not addressed<br>Adequately addressed / Not reported<br>Poorly addressed / Not applicable |
| 2.9 | All relevant outcomes are measured in a valid way.<br>Comments: | Well covered / Not addressed<br>Adequately addressed / Not reported<br>Poorly addressed / Not applicable |
| 2.10 | What percentage of the individuals or clusters recruited into the study dropped out before the study was completed?<br>Comments:<br>*Were reasons given for withdrawals/drop-outs?* | |
| 2.11 | All the subjects are analysed in the groups to which they were randomly allocated (often referred to as intention to treat analysis).<br>Comments: | Well covered / Not addressed<br>Adequately addressed / Not reported<br>Poorly addressed / Not applicable |
| 2.12 | Where the study is carried out at more than one site, results are comparable for all sites.<br>Comments: | Well covered / Not addressed<br>Adequately addressed / Not reported<br>Poorly addressed / Not applicable |

| SECTION 3: OVERALL ASSESSMENT OF THE STUDY | | | | | |
|---|---|---|---|---|---|
| 3.1 | How well was the study done to minimise bias?<br>Comments: | | ++ | + | - |
| 3.2 | If coded as + or –, what is the likely direction in which bias might affect the study results?<br>Comments: | | | | |
| 3.3 | Taking into account clinical considerations, your evaluations of the methodology used, and the statistical power of the study, are you certain that the overall effect is due to the study intervention?<br>Comments: | | Yes | No | |
| 3.4 | Are the results of this study directly applicable to the patient group targeted?<br>Comments: | | Yes | No | |
| 3.5 | Is the analysis appropriate (e.g. does it align with the research question)?<br>Comments: | | Yes | No | |
| 3.6 | Main strengths | | | | |
| 3.7 | Main weaknesses | | | | |
| 3.8 | References to be checked: | | | | |
| 3.9 | References of methodological or other interest: | | | | |

| | |
|---|---|
| | |

| ADDITIONAL COMMENTS |
|---|
| What percentage of selected individuals agreed to participate (i.e. response rate)?<br><br>Is non-randomized treatment allocation adequate?<br><br>Comment on the length and completeness of follow-up:<br><br>Can we generalize the results? (external validity) |

# Methodology Checklist 3: Cohort studies

SIGN

## SECTION 1: STUDY IDENTIFICATION

| | | | |
|---|---|---|---|
| ID: | First author: | Year: | Journal: |
| Title: | | | |
| Objectives: | | | |
| Language: | | | |
| Country: | Admissible Inadmissible | Checklist completed by: | |

## SECTION 2: INTERNAL VALIDITY

| *In a well conducted cohort study:* | | *In this study the criterion is:* | |
|---|---|---|---|
| 2.1 | The study addresses an appropriate and clearly focused question.<br>Comments: | Well covered<br>Adequately addressed<br>Poorly addressed | Not addressed<br>Not reported<br>Not applicable |
| 2.2 | The definition of MTBI is clear.<br>Comments: | Well covered<br>Adequately addressed<br>Poorly addressed | Not addressed<br>Not reported<br>Not applicable |
| **SELECTION OF SUBJECTS** | | | |
| 2.3 | The groups being studied are selected from the same source population and are comparable in all respects other than the factor under investigation.<br>Comments: | Well covered<br>Adequately addressed<br>Poorly addressed | Not addressed<br>Not reported<br>Not applicable |
| 2.4 | The study indicates how many of the people asked to take part did participate, in each of the groups being studied.<br>Comments: | Well covered<br>Adequately addressed<br>Poorly addressed | Not addressed<br>Not reported<br>Not applicable |

| | | |
|---|---|---|
| 2.5 | The likelihood that some eligible subjects might have the outcome at the time of enrolment is assessed and taken into account in the design and/or analysis.<br>Comments: | Well covered<br>Adequately addressed<br>Poorly addressed<br>Not addressed<br>Not reported<br>Not applicable |
| 2.6 | What percentage of individuals or clusters recruited into the study dropped out before the study was completed?<br>Comments:<br>*Were reasons for drop-out/withdrawal given?* | |
| 2.7 | *Comparison is made between full participants and those lost to follow up, by exposure status.*<br>*Comments:* | Well covered<br>Adequately addressed<br>Poorly addressed<br>Not addressed<br>Not reported<br>Not applicable |
| ASSESSMENT | | |
| 2.8 | The outcomes are clearly defined.<br>Comments: | Well covered<br>Adequately addressed<br>Poorly addressed<br>Not addressed<br>Not reported<br>Not applicable |
| 2.9 | The assessment of outcome is made blind to exposure status.<br>Comments: | Well covered<br>Adequately addressed<br>Poorly addressed<br>Not addressed<br>Not reported<br>Not applicable |
| 2.10 | Where blinding was not possible, there is some recognition that knowledge of exposure status could have influenced the assessment of outcome.<br>Comments: | Well covered<br>Adequately addressed<br>Poorly addressed<br>Not addressed<br>Not reported<br>Not applicable |

| | | |
|---|---|---|
| 2.11 | The measure of assessment of exposure is reliable.<br>Comments: | Well covered / Adequately addressed / Poorly addressed / Not addressed / Not reported / Not applicable |
| 2.12 | The measure of assessment of exposure is valid.<br>Comments: | Well covered / Adequately addressed / Poorly addressed / Not addressed / Not reported / Not applicable |
| 2.13 | Evidence from other sources is used to demonstrate that the method of outcome assessment is reliable.<br>Comments: | Well covered / Adequately addressed / Poorly addressed / Not addressed / Not reported / Not applicable |
| 2.14 | Evidence from other sources is used to demonstrate that the method of outcome assessment is valid.<br>Comments: | Well covered / Adequately addressed / Poorly addressed / Not addressed / Not reported / Not applicable |
| 2.15 | Exposure level or prognostic factor is assessed more than once.<br>Comments: | Well covered / Adequately addressed / Poorly addressed / Not addressed / Not reported / Not applicable |
| CONFOUNDING | | |
| 2.16 | The main potential confounders are identified and taken into account adequately in the design and analysis.<br>Comments: | Well covered / Adequately addressed / Poorly addressed / Not addressed / Not reported / Not applicable |
| STATISTICAL ANALYSIS | | |
| 2.17 | Have confidence intervals been provided?<br>Comments: | Yes / No |

| | | |
|---|---|---|
| | | |

## SECTION 3: OVERALL ASSESSMENT OF THE STUDY

| | | |
|---|---|---|
| 3.1 | How well was the study done to minimise the risk of bias or confounding, and to establish a causal relationship between exposure and effect?<br>Comments: | ++    +    - |
| 3.2 | If coded as + or -, what is the likely direction in which bias might affect the study results?<br>Comments: | |
| 3.3 | Taking into account clinical considerations, your evaluations of the methodology used, and the statistical power of the study, are you certain that the overall effect is due to the exposure being investigated?<br>Comments: | Yes    No |
| 3.4 | Are the results of this study directly applicable to the patient group targeted in this study?<br>Comments: | Yes    No |
| 3.5 | Phase of study | 1 – Exploratory studies (hypothesis generating)<br>2 – Exploratory studies (employ matching, stratification or multivariable analyses)<br>3 – Confirmatory studies that test a priori hypotheses |
| 3.6 | Main strengths | |
| 3.7 | Main weaknesses | |
| 3.8 | References to be checked: | |

| | |
|---|---|
| | |
| 3.9 | References of methodological or other interest: |

| Additional Comments |
|---|
| Type of cohort study? Prospective Retrospective Mixed<br><br>Comment on the length and completeness of follow-up:<br><br>Is it likely that subjects received an unintended exposure (contamination or co-intervention) that may influence results?<br><br>Can we generalize the results? (external validity) |

SIGN

# Methodology Checklist 1: Systematic Reviews and Meta-analyses

## SECTION 1: STUDY IDENTIFICATION

| ID: | First author: | Year: | Journal: |
|---|---|---|---|
| Title: | | | |
| Objectives: | | | |
| Language: | | | |
| Country: | Admissible Inadmissible | | Checklist completed by: |

## SECTION 2: INTERNAL VALIDITY

| *In a well conducted systematic review* | | *In this study this criterion is:* | |
|---|---|---|---|
| 2.1 | The study addresses an appropriate and clearly focused question.<br>Comments: | Well covered<br>Adequately addressed<br>Poorly addressed | Not addressed<br>Not reported<br>Not applicable |
| 2.2 | The definition of MTBI is clear.<br>Comments: | Well covered<br>Adequately addressed<br>Poorly addressed | Not addressed<br>Not reported<br>Not applicable |
| 2.3 | A description of the methodology used is included.<br>Comments: | Well covered<br>Adequately addressed<br>Poorly addressed | Not addressed<br>Not reported<br>Not applicable |
| 2.4 | The literature search is sufficiently rigorous to identify all the relevant studies.<br>Comments: | Well covered<br>Adequately addressed<br>Poorly addressed | Not addressed<br>Not reported<br>Not applicable |
| 2.5 | Study quality is assessed and taken into account.<br>Comments: | Well covered<br>Adequately addressed<br>Poorly addressed | Not addressed<br>Not reported<br>Not applicable |
| 2.6 | There are enough similarities between the studies selected to make combining them reasonable.<br>Comments: | Well covered<br>Adequately addressed | Not addressed<br>Not reported<br>Not applicable |

| | | |
|---|---|---|
| | | Poorly addressed |

## SECTION 3: OVERALL ASSESSMENT OF THE STUDY

| | | | |
|---|---|---|---|
| 3.1 | How well was the study done to minimise bias?<br><br>Comments: | ++ + - | |
| 3.2 | If coded as +, or – what is the likely direction in which bias might affect the study results?<br><br>Comments: | | |
| 3.3 | Are the results of this study directly applicable to the objectives of this systematic review?<br><br>Comments: | | Yes No |
| 3.4 | Main strengths | | |
| 3.5 | Main weaknesses | | |
| 3.6 | References to be checked: | | |
| 3.7 | References of methodological or other interest: | | |

### ADDITIONAL COMMENTS

How adequately was the synthesis done?

Can we generalize the results (external validity)?

# Methodology Checklist 4: Case-control studies

SIGN

## SECTION 1: STUDY IDENTIFICATION

| | | | |
|---|---|---|---|
| ID: | First author: | Year: | Journal: |
| Title: | | | |
| Objectives: | | | |
| Language: | | | |
| Country: | Admissible Inadmissible | | Checklist completed by: |

## SECTION 2: INTERNAL VALIDITY

| | ***In a well conducted case-control study:*** | ***In this study the criterion is:*** | |
|---|---|---|---|
| 2.1 | The study addresses an appropriate and clearly focused question.<br>Comments: | Well covered<br>Adequately addressed<br>Poorly addressed | Not addressed<br>Not reported<br>Not applicable |
| 2.2 | The definition of MTBI is clear.<br>Comments: | Well covered<br>Adequately addressed<br>Poorly addressed | Not addressed<br>Not reported<br>Not applicable |
| SELECTION OF SUBJECTS | | | |
| 2.3 | The cases and controls are taken from comparable populations.<br>Comments: | Well covered<br>Adequately addressed<br>Poorly addressed | Not addressed<br>Not reported<br>Not applicable |
| 2.4 | The same exclusion criteria are used for both cases and controls.<br>Comments: | Well covered<br>Adequately addressed<br>Poorly addressed | Not addressed<br>Not reported<br>Not applicable |
| 2.5 | What percentage of each group (cases and controls) participated in the study?<br>Comments: | Cases:<br>Controls: | |

| | | | |
|---|---|---|---|
| | | | |
| 2.6 | Comparison is made between participants and non-participants to establish their similarities or differences.<br>Comments: | Well covered<br>Adequately addressed<br>Poorly addressed | Not addressed<br>Not reported<br>Not applicable |
| 2.7 | Cases are clearly defined and differentiated from controls.<br>Comments: | Well covered<br>Adequately addressed<br>Poorly addressed | Not addressed<br>Not reported<br>Not applicable |
| 2.8 | *It is clearly established that controls are non-cases.*<br>*Comments:* | Well covered<br>Adequately addressed<br>Poorly addressed | Not addressed<br>Not reported<br>Not applicable |
| ASSESSMENT | | | |
| 2.9 | Measures will have been taken to prevent knowledge of primary exposure influencing case ascertainment.<br>Comments: | Well covered<br>Adequately addressed<br>Poorly addressed | Not addressed<br>Not reported<br>Not applicable |
| 2.10 | Exposure status is measured in a reliable way.<br>Comments: | Well covered<br>Adequately addressed<br>Poorly addressed | Not addressed<br>Not reported<br>Not applicable |
| 2.11 | Exposure status is measured in a valid way.<br>Comments: | Well covered<br>Adequately addressed<br>Poorly addressed | Not addressed<br>Not reported<br>Not applicable |
| CONFOUNDING | | | |
| 2.12 | The main potential confounders are identified and taken into account in the design and analysis.<br>Comments: | Well covered<br>Adequately addressed<br>Poorly addressed | Not addressed<br>Not reported<br>Not applicable |

| | | |
|---|---|---|
| | | |

| Statistical Analysis | | |
|---|---|---|
| 2.13 | Confidence intervals are provided.<br>Comments: | Yes No |

| SECTION 3: OVERALL ASSESSMENT OF THE STUDY | | |
|---|---|---|
| 3.1 | *How well was the study done to minimise the risk of bias or confounding?*<br>*Comments:* | ++ + - |
| 3.2 | If coded as + or –, what is the likely direction in which bias might affect the study results?<br>Comments: | |
| 3.3 | Taking into account clinical considerations, your evaluations of the methodology used, and the statistical power of the study, are you certain that the overall effect is due to the exposure being investigated?<br>Comments: | Yes No |

| | | |
|---|---|---|
| 3.4 | Phase of study | 1 – Exploratory studies (hypothesis generating)<br>2 – Exploratory studies (employ matching, stratification or multivariable analyses)<br>3 – Confirmatory studies that test a priori hypotheses |
| 3.5 | Main strengths | |
| 3.6 | Main weaknesses | |
| 3.7 | References to be checked: | |
| 3.8 | References of methodological or other interest: | |

| Additional Comments |
| --- |
| Can we generalize the results? (external validity) |
