# Supplementary material for: Protocol for a systematic review of prognosis after mild traumatic brain injury: an update of the WHO Collaborating Centre Task Force findings
Source: Syst Rev. 2012 Feb 23;1:17. doi: 10.1186/2046-4053-1-17 (PMC3351713; doi:10.1186/2046-4053-1-17)
Supplement: Additional file 3 — Scottish Intercollegiate Guidelines Network (SIGN) notes. This file contains modified SIGN notes used to aid reviewers in completing the SIGN checklists. [file 2046-4053-1-17-S3.PDF]

NOTES ON THE USE OF METHODOLOGY CHECKLIST: RANDOMIZED CONTROLLED TRIALS

SECTION 1 identifies the study, the reviewer, and the objective(s) it is expected to address. The reviewer is asked to consider a series of aspects of RCT design and to make a judgment as to how well the current study meets this criterion. Each relates to an aspect of methodology that research has shown makes a significant difference to the conclusions of a study.

For each question in this section you should use one of the following to indicate how well it has been addressed in the study:

- Well covered
- Adequately addressed
- Poorly addressed
- Not addressed *(i.e. not mentioned, or indicates that this aspect of study design was ignored)*
- Not reported *(i.e. mentioned, but insufficient detail to allow assessment to be made)*
- Not applicable.

2.1 THE STUDY ADDRESSES AN APPROPRIATE AND CLEARLY FOCUSED QUESTION

Unless a clear and well defined question is specified, it will be difficult to assess how well the study has met its objectives or how relevant it is to the question you are trying to answer on the basis of its conclusions.

2.2 THE DEFINITION OF MTBI IS CLEAR.

Our case definition of MTBI is:

"MTBI is an acute brain injury resulting from mechanical energy to the head from external physical forces. Operational criteria for clinical identification include: (i) one or more of the following: confusion or disorientation, loss of consciousness for 30 minutes or less, post-traumatic amnesia for less than 24 hours, and/or other transient neurological abnormalities such as focal signs, seizure, and intracranial lesion not requiring surgery; (ii) Glasgow Coma Scale score of 13 – 15 after 30 minutes post-injury or later upon presentation for healthcare. These manifestations of MTBI must not be due to drugs, alcohol, medications, caused by other injuries or treatment for other injuries (e.g. systemic injuries, facial injuries or intubation), caused by other problems (e.g. psychological trauma, language barrier or coexisting medical conditions) or caused by penetrating craniocerebral injury." (Carroll et al., J Rehabil Med, 2004, p. 115).

Persons with skull fractures included if they fit this case definition.

Additional from CDC: Abbreviated Injury Severity Scale (AIS) score of 2 for the head region; and the following ICD-9-CM codes.

ICD-9-CM First Four Digits =	ICD-9-CM Fifth Digit =
800.0, 800.5, 801.0, 801.5,<br>803.0, 803.5, 804.0, 804.5,<br>850.0, 850.1, 850.5 or 850.9	0, 1, 2, 6, 9, or Missing
854.0	1, 2, 6, 9, or Missing

959.0*	1

*The current inclusion of code 959.01 is provisional.

2.3 THE ASSIGNMENT OF SUBJECTS TO TREATMENT GROUPS IS RANDOMIZED

Random allocation of patients to receive one or other of the treatments under investigation, or to receive either treatment or placebo, is fundamental to this type of study. **If there is no indication of randomization, the study should be rejected.** If the description of randomization is poor, the study should be given a lower quality rating. Processes such as alternate allocation, allocation by date of birth, or day of the week attending a clinic are not true randomization processes and it is easy for a researcher to work out which patients received which treatment. These studies should therefore be classed as Controlled Clinical Trials rather than RCTs.

2.4 AN ADEQUATE CONCEALMENT METHOD IS USED

Allocation concealment refers to the process used to ensure that researchers are unaware which group patients are being allocated to at the time they enter the study. Research has shown that where allocation concealment is inadequate, investigators can overestimate the effect of interventions by up to 40%. Centralized allocation, computerized allocation systems, or the use of coded identical containers would all be regarded as adequate methods of concealment, and may be taken as indicators of a well conducted study. If the method of concealment used is regarded as poor, or relatively easy to subvert, the study must be given a lower quality rating, and can be rejected if the concealment method is seen as inadequate.

2.5 SUBJECTS AND INVESTIGATORS ARE KEPT 'BLIND' TO TREATMENT ALLOCATION

Blinding refers to the process whereby people are kept unaware of which treatment an individual patient has been receiving when they are assessing the outcome for that patient. It can be carried out up to three levels. Single blinding is where patients are unaware of which treatment they are receiving. In double blind studies neither the doctor nor the patient knows which treatment is being given. In very rare cases studies may be triple blinded, where neither patients, doctors, nor those conducting the analysis are aware of which patients received which treatment. The higher the level of blinding, the lower the risk of bias in the study.

2.6 THE TREATMENT AND CONTROL GROUPS ARE SIMILAR AT THE START OF THE TRIAL

Patients selected for inclusion in a trial must be as similar as possible. The study should report any significant differences in the composition of the study groups in relation to gender mix, age, stage of disease (if appropriate), social background, ethnic origin, or comorbid conditions. These factors may be covered by inclusion and exclusion criteria, rather than being reported directly. Failure to address this question, or the use of inappropriate groups, should lead to the study being downgraded.

2.7 THE ONLY DIFFERENCE BETWEEN THE GROUPS IS THE TREATMENT UNDER INVESTIGATION

If some patients received additional treatment, even if of a minor nature or consisting of advice and counseling rather than a physical intervention, this treatment is a potential confounding factor that may invalidate the results. **If groups were *not* treated equally, the study should be rejected unless no other evidence is available.** If the study is used as evidence it should be treated with caution.

2.8 ALL RELEVANT OUTCOMES ARE MEASURED IN A RELIABLE WAY

Where outcome measures require any degree of subjectivity, some evidence should be provided that the measures used are reliable prior to their use in the study.

2.9 ALL RELEVANT OUTCOMES ARE MEASURED IN A VALID WAY

The primary outcome measures used should be clearly stated in the study. **If the outcome measures are not stated, or the study bases its main conclusions on secondary outcomes, the study should be rejected.** Where outcome measures require any degree of subjectivity, some evidence should be provided that the measures used have been validated prior to their use in the study.

2.10 WHAT PERCENTAGE OF THE INDIVIDUALS OR CLUSTERS RECRUITED INTO THE STUDY DROPPED OUT BEFORE THE STUDY WAS COMPLETED?

The number of patients that drop out of a study should give concern if the number is very high. Conventionally, a 20% drop out rate is regarded as acceptable, but this may vary. Some regard should be paid to why patients dropped out, as well as how many. It should be noted that the drop out rate may be expected to be higher in studies conducted over a long period of time. A higher drop out rate will normally lead to downgrading, rather than rejection of a study.

2.11 ALL THE SUBJECTS ARE ANALYSED IN THE GROUPS TO WHICH THEY WERE RANDOMLY ALLOCATE (INTENTION TO TREAT ANALYSIS)

In practice, it is rarely the case that all patients allocated to the intervention group receive the intervention throughout the trial, or that all those in the comparison group do not. Patients may refuse treatment, or contra-indications arise that lead them to be switched to the other group. If the comparability of groups through randomization is to be maintained, however, patient outcomes must be analyzed according to the group to which they were originally allocated irrespective of the treatment they actually received. (This is known as intention to treat analysis.) If it is clear that analysis was not on an intention to treat basis, the study may be rejected. If there is little other evidence available, the study may be included but should be evaluated as if it were a non-randomized cohort study.

2.12 WHERE THE STUDY IS CARRIED OUT AT MORE THAN ONE SITE, RESULTS ARE COMPARABLE FOR ALL SITES

In multi-site studies, confidence in the results should be increased if it can be shown that similar results were obtained at the different participating centres.

SECTION 3 relates to the overall assessment of the paper. It starts by rating the methodological quality of the study, based on your responses in Section 2 and using the following coding system:

**++**	**All or most** of the criteria have been fulfilled. Where they have not been fulfilled the conclusions of the study or review are thought very unlikely to alter.
**+**	**Some** of the criteria have been fulfilled. Those criteria that have not been fulfilled or not adequately described are thought unlikely to alter the conclusions.
**-**	**Few or no** criteria fulfilled. The conclusions of the study are thought likely or very likely to alter.

The code allocated here, coupled with the study phase, will decide the **level of evidence** that this study provides. Phase 3 studies will be given the most weight.

The aim of the other questions in this section is to allow you to respond to other issues regarding methodology that are not present elsewhere in the form (and may help us in synthesizing our results) as well as summarize your view of the quality of this study and its applicability to the patient group targeted.

Scottish Intercollegiate Guidelines Network,
Elliott House, 8-10 Hillside Crescent,
Edinburgh EH7 5EA
Web contact duncan.service@nhs.net
Last modified 21/04/11
© SIGN 2001-2011

SIGN

NOTES ON THE USE OF METHODOLOGY CHECKLIST: COHORT STUDIES

The studies covered by this checklist are designed to answer questions of the type “What are the effects of this exposure?” It relates to studies that compare a group of people with a particular exposure with another group who either have not had the exposure, or have a different level of exposure. Cohort studies may be prospective (where the exposure is defined and subjects selected before outcomes occur), or retrospective (where exposure is assessed after the outcome is known, usually by the examination of medical records). Retrospective studies are generally regarded as a weaker design, and should not receive a "++" rating.

SECTION 1 identifies the study, the reviewer, and the objective(s) it is expected to address. The reviewer is asked to consider a series of aspects of cohort study design and to make a judgment as to how well the current study meets this criterion. Each relates to an aspect of methodology that research has shown to be likely to influence the conclusions of a study.

Because of the potential complexity and subtleties of the design of this type of study, there are comparatively few criteria that automatically rule out use of a study as evidence. It is more a matter of increasing confidence in the strength of association between exposure and outcome by identifying how many aspects of good study design are present, and how well they have been tackled. A study that fails to address or report on more than one or two of the questions addressed below should almost certainly be rejected.

For each question in this section you should use one of the following to indicate how well it has been addressed in the study:

- Well covered
- Adequately addressed
- Poorly addressed
- Not addressed (i.e. not mentioned, or indicates that this aspect of study design was ignored)
- Not reported (i.e. mentioned, but insufficient detail to allow assessment to be made)
- Not applicable.

2.1 THE STUDY ADDRESSES AN APPROPRIATE AND CLEARLY FOCUSED QUESTION.

Unless a clear and well defined question is specified, it will be difficult to assess how well the study has met its objectives or how relevant it is to the question you are trying to answer on the basis of its conclusions.

2.2 THE DEFINITION OF MTBI IS CLEAR.

Our case definition of MTBI is:

“MTBI is an acute brain injury resulting from mechanical energy to the head from external physical forces. Operational criteria for clinical identification include: (i) one or more of the following: confusion or disorientation, loss of consciousness for 30 minutes or less, post-traumatic amnesia for less than 24 hours, and/or other transient neurological abnormalities such as focal signs, seizure, and intracranial lesion not requiring surgery; (ii) Glasgow Coma Scale score of 13 – 15 after 30 minutes post-injury or later upon presentation for healthcare. These manifestations of MTBI must not be due to drugs, alcohol, medications, caused by other injuries or treatment for other injuries (e.g. systemic injuries, facial injuries or intubation), caused by other problems (e.g. psychological trauma, language barrier or coexisting medical conditions) or caused by penetrating craniocerebral injury.” (Carroll et al., J Rehabil Med, 2004, p. 115).

Persons with skull fractures included if they fit this case definition.

Additional from CDC: Abbreviated Injury Severity Scale (AIS) score of 2 for the head region; and the following ICD-9-CM codes.

ICD-9-CM First Four Digits =	ICD-9-CM Fifth Digit =
800.0, 800.5, 801.0, 801.5, 803.0, 803.5, 804.0, 804.5, 850.0, 850.1, 850.5 or 850.9	0, 1, 2, 6, 9, or Missing
854.0	1, 2, 6, 9, or Missing
959.0*	1

*The current inclusion of code 959.01 is provisional.

2.3 THE GROUPS BEING STUDIED ARE SELECTED FROM THE SAME SOURCE POPULATION AND ARE COMPARABLE IN ALL RESPECTS OTHER THAN THE FACTOR UNDER INVESTIGATION.

It is important that the groups selected for comparison are as similar as possible in all characteristics except for their exposure status, or the presence of specific prognostic factors or prognostic markers relevant to the study in question. **If the study does not include clear definitions of the source population and eligibility criteria for participants it should be rejected.**

2.4 THE STUDY INDICATES HOW MANY OF THE PEOPLE ASKED TO TAKE PART DID PARTICIPATE, IN EACH OF THE GROUPS BEING STUDIED.

The participation rate is defined as the number of study participants divided by the number of eligible subjects, and should be calculated separately for each group of the study. A large difference in participation rate between the different groups of the study indicates that a significant degree of selection bias may be present, and the study results should be treated with considerable caution.

2.5 THE LIKELIHOOD THAT SOME ELIGIBLE SUBJECTS MIGHT HAVE THE OUTCOME AT THE TIME OF ENROLMENT IS ASSESSED AND TAKEN INTO ACCOUNT IN THE DESIGN AND/OR ANALYSIS.

If some of the eligible subjects, particularly those in the unexposed group, already have the outcome at the start of the trial the final result will be biased. A well conducted study will attempt to estimate the likelihood of this occurring, and take it into account in the analysis through the use of sensitivity studies or other methods.

2.6 WHAT PERCENTAGE OF INDIVIDUALS OR CLUSTERS RECRUITED INTO THE STUDY DROPPED OUT BEFORE THE STUDY WAS COMPLETED?

The number of patients that drop out of a study should give concern if the number is very high. Conventionally, a 20% drop out rate is regarded as acceptable, but in observational studies conducted over a lengthy period of time a higher drop out rate is to be expected. A decision on whether to downgrade or reject a study because of a high drop out rate is a matter of judgment based on the reasons why people dropped out, and whether drop out rates were comparable in the exposed and unexposed groups. Reporting of efforts to follow up participants that dropped out may be regarded as an indicator of a well conducted study.

2.7 COMPARISON IS MADE BETWEEN FULL PARTICIPANTS AND THOSE LOST TO FOLLOW-UP, BY EXPOSURE STATUS.

For valid study results, it is essential that the study participants are truly representative of the source population. It is always possible that participants who dropped out of the study will differ in some significant way from those who remained part of the study throughout. A well conducted study will attempt to identify any such differences between full and partial participants in both the exposed and unexposed groups. Any indication that differences exist, should lead to the study results being treated with caution.

2.8 THE OUTCOMES ARE CLEARLY DEFINED.

Once enrolled in the study, participants should be followed until specified end points or outcomes are reached. In a study of the effect of exercise on the death rates from heart disease in middle aged men, for example, participants might be followed up until death, or until reaching a predefined age. **If outcomes and the criteria used for measuring them are not clearly defined, the study should be rejected.**

2.9 THE ASSESSMENT OF OUTCOME IS MADE BLIND TO EXPOSURE STATUS.

If the assessor is blinded to which participants received the exposure, and which did not, the prospects of unbiased results are significantly increased. Studies in which this is done should be rated more highly than those where it is not done, or not done adequately.

2.10 WHERE BLINDING WAS NOT POSSIBLE, THERE IS SOME RECOGNITION THAT KNOWLEDGE OF EXPOSURE STATUS COULD HAVE INFLUENCED THE ASSESSMENT OF OUTCOME.

Blinding is not possible in many cohort studies. In order to assess the extent of any bias that may be present, it may be helpful to compare process measures used on the participant groups - e.g. frequency of observations, who carried out the observations, the degree of detail and completeness of observations. If these process measures are comparable between the groups, the results may be regarded with more confidence.

2.11 THE MEASURE OF ASSESSMENT OF EXPOSURE IS RELIABLE.

Clearly described, reliable measures should increase the confidence in the quality of the study.

2.12 THE MEASURE OF ASSESSMENT OF EXPOSURE IS VALID.

A well conducted study should indicate how the degree of exposure or presence of prognostic factors or markers was assessed. Whatever measures are used must be sufficient to establish clearly that participants have or have not received the exposure under investigation and the extent of such exposure, or that they do or do not possess a particular prognostic marker or factor. Clearly described, valid measures should increase the confidence in the quality of the study.

2.13 EVIDENCE FROM OTHER SOURCES IS USED TO DEMONSTRATE THAT THE METHOD OF OUTCOME ASSESSMENT IS RELIABLE.

Where outcome measures require any degree of subjectivity, some evidence should be provided that the measures used are reliable prior to their use in the study.

2.14 EVIDENCE FROM OTHER SOURCES IS USED TO DEMONSTRATE THAT THE METHOD OF OUTCOME ASSESSMENT IS VALID.

The primary outcome measures used should be clearly stated in the study. **If the outcome measures are not stated, or the study bases its main conclusions on secondary outcomes, the study should be rejected.** Where outcome measures require any degree of subjectivity, some evidence should be provided that the measures used have been validated prior to their use in the study.

2.15 EXPOSURE LEVEL OR PROGNOSTIC FACTOR IS ASSESSED MORE THAN ONCE.

Confidence in data quality should be increased if exposure level is measured more than once in the course of the study. Independent assessment by more than one investigator is preferable.

2.16 THE MAIN POTENTIAL CONFOUNDERS ARE IDENTIFIED AND TAKEN INTO ACCOUNT ADEQUATELY IN THE DESIGN AND ANALYSIS.

Confounding is the distortion of a link between exposure and outcome by another factor that is associated with both exposure and outcome. The possible presence of confounding factors is one of the principal reasons why observational studies are not more highly rated as a source of evidence. The report of the study should indicate which potential confounders have been considered, and how they have been assessed or allowed for in the

analysis. Clinical judgment should be applied to consider whether all likely confounders have been considered. If the measures used to address confounding are considered inadequate, the study should be downgraded or rejected, depending on how serious the risk of confounding is considered to be. **A study that does not address the possibility of confounding should be rejected.**

2.17 HAVE CONFIDENCE INTERVALS BEEN PROVIDED?

Confidence limits are the preferred method for indicating the precision of statistical results, and can be used to differentiate between an inconclusive study and a study that shows no effect. Studies that report a single value with no assessment of precision should be treated with extreme caution.

SECTION 3 relates to the overall assessment of the paper. It starts by rating the methodological quality of the study, based on your responses in Section 2 and using the following coding system:

**++**	**All or most** of the criteria have been fulfilled.<br>Where they have not been fulfilled the conclusions of the study or review are thought *very unlikely* to alter.
**+**	**Some** of the criteria have been fulfilled.<br>Those criteria that have not been fulfilled or not adequately described are thought *unlikely* to alter the conclusions.
**-**	**Few or no** criteria fulfilled<br>The conclusions of the study are thought *likely or very likely* to alter.

The code allocated here, coupled with the study phase, will decide the **level of evidence** that this study provides. Phase 3 studies will be given the most weight.

3.5 PHASES OF STUDY

Phase 1: Studies are exploratory, hypothesis-generating studies characterised by descriptive explorations and demonstration of crude (unadjusted) associations.

Phase 2: Studies are also exploratory, but employ matching, stratification or multivariable analyses to identify independent prognostic factors, or employ multivariable models to predict outcomes.

Phase 3: Studies are confirmatory studies that test a priori hypotheses about one or more factors as independent predictors of outcomes, or groups of variables for clinical prediction. These studies include explicit control for confounding factors, or validate a clinical prediction rule.

The aim of the other questions in this section is to allow you to respond to other issues regarding methodology that are not present elsewhere in the form (and may help us in synthesizing our results) as well as summarize your view of the quality of this study and its applicability to the patient group targeted.

Scottish Intercollegiate Guidelines Network,
Elliott House, 8-10 Hillside Crescent,
Edinburgh EH7 5EA
Web contact duncan.service@nhs.net
Last modified 21/04/11
© SIGN 2001-2011

NOTES ON THE USE OF METHODOLOGY CHECKLIST: SYSTEMATIC REVIEWS AND META-ANALYSES

SECTION 1 identifies the study, the reviewer, and the objective(s) it is expected to address. The reviewer is asked to consider a series of aspects of study design and to make a judgment as to how well the current study meets each criterion. Each relates to an aspect of methodology that research has shown to be likely to influence the conclusions of a study.

For each question in this section you should use one of the following to indicate how well it has been addressed in the study:

- Well covered
- Adequately addressed
- Poorly addressed
- Not addressed (i.e. not mentioned, or indicates that this aspect of study design was ignored)
- Not reported (i.e. mentioned, but insufficient detail to allow assessment to be made)
- Not applicable.

2.1 THE STUDY ADDRESSES AN APPROPRIATE AND CLEARLY FOCUSED QUESTION.

Unless a clear and well defined question is specified in the report of the review, it will be difficult to assess how well it has met its objectives or how relevant it is to the question you are trying to answer on the basis of the conclusions.

2.2 THE DEFINITION OF MTBI IS CLEAR.

Our case definition of MTBI is:

"MTBI is an acute brain injury resulting from mechanical energy to the head from external physical forces. Operational criteria for clinical identification include: (i) one or more of the following: confusion or disorientation, loss of consciousness for 30 minutes or less, post-traumatic amnesia for less than 24 hours, and/or other transient neurological abnormalities such as focal signs, seizure, and intracranial lesion not requiring surgery; (ii) Glasgow Coma Scale score of 13 – 15 after 30 minutes post-injury or later upon presentation for healthcare. These manifestations of MTBI must not be due to drugs, alcohol, medications, caused by other injuries or treatment for other injuries (e.g. systemic injuries, facial injuries or intubation), caused by other problems (e.g. psychological trauma, language barrier or coexisting medical conditions) or caused by penetrating craniocerebral injury." (Carroll et al., J Rehabil Med, 2004, p. 115).

Persons with skull fractures included if they fit this case definition.

Additional from CDC: Abbreviated Injury Severity Scale (AIS) score of 2 for the head region; and the following ICD-9-CM codes.

ICD-9-CM First Four Digits =	ICD-9-CM Fifth Digit =
800.0, 800.5, 801.0, 801.5, 803.0, 803.5, 804.0, 804.5, 850.0, 850.1, 850.5 or 850.9	0, 1, 2, 6, 9, or Missing
854.0	1, 2, 6, 9, or Missing

959.0*	1

*The current inclusion of code 959.01 is provisional.

2.3 A DESCRIPTION OF THE METHODOLOGY USED IS INCLUDED.

One of the key distinctions between a systematic review and a general review is the systematic methodology used. A systematic review should include a detailed description of the methods used to identify and evaluate individual studies. If this description is not present, it is not possible to make a thorough evaluation of the quality of the review, and it should be rejected or considered low level evidence.

2.4 THE LITERATURE SEARCH IS SUFFICIENTLY RIGOROUS TO IDENTIFY ALL THE RELEVANT STUDIES.

A systematic review based on a limited literature search – e.g. one limited to Medline only – is likely to be heavily biased. A well conducted review should as a minimum look at Embase and Medline, and from the late 1990s onward, as well as the Cochrane Library. Any indication that hand searching of key journals, or follow up of reference lists of included studies were carried out in addition to electronic database searches can be taken as evidence of a well conducted review.

2.5 STUDY QUALITY IS ASSESSED AND TAKEN INTO ACCOUNT.

A well conducted systematic review should have used clear criteria to assess whether individual studies had been well conducted before deciding whether to include or exclude them. If there is no indication of such an assessment, the individual papers included in the review must be obtained and their methodology evaluated.

2.6 THERE ARE ENOUGH SIMILARITIES BETWEEN THE STUDIES SELECTED TO MAKE COMBINING THEM REASONABLE.

Studies covered by a systematic review should be selected using clear inclusion criteria. These criteria should include, either implicitly or explicitly, the question of whether the selected studies can legitimately be compared. It should be clearly ascertained, for example, that the populations covered by the studies are comparable; that the methods used in the investigations are the same; that the outcome measures are comparable; and the variability in effect sizes between studies is not greater than would be expected by chance alone.

SECTION 3 relates to the overall assessment of the paper. Question 3.1 asks you to rate the methodological quality of the study, based on your responses in Section 2 and using the following coding system:

++	All or most of the criteria have been fulfilled. Where they have not been fulfilled the conclusions of the study or review are thought very unlikely to alter.
+	Some of the criteria have been fulfilled. Those criteria that have not been fulfilled or not adequately described are thought unlikely to alter the conclusions.
-	Few or no criteria fulfilled. The conclusions of the study are thought likely or very likely to alter.

The code allocated here, coupled with the study phase, will decide the level of evidence that this study provides. Phase 3 studies will be given the most weight.

QUESTION 3.2 asks you to indicate whether a review with poor or relatively poor methodology is likely to overstate or understate any effect identified.

Scottish Intercollegiate Guidelines Network,
Elliott House, 8-10 Hillside Crescent,
Edinburgh EH7 5EA
Web contact duncan.service@nhs.net
Last modified 21/04/11
© SIGN 2001-2011

NOTES ON THE USE OF METHODOLOGY CHECKLIST 4: CASE-CONTROL STUDIES

The studies covered by this checklist are designed to answer questions of the type “What are the factors that caused this event?”, and involve comparison of individuals with an outcome with other individuals from the same population who do not have the outcome. These studies start after the outcome of an event, and can be used to assess multiple causes of a single event. They are generally used to assess the causes of a new problem, but may also be useful for the evaluation of population based interventions such as screening.

SECTION 1 identifies the study, the reviewer, and the objective(s) it is expected to address. The reviewer is asked to consider a series of aspects of case control study design and to make a judgement as to how well the current study meets this criterion. Each relates to an aspect of methodology that research has shown makes a significant difference to the conclusions of a study.

Case-control studies need to be very carefully designed, and the complexity of their design is often not appreciated by investigators, leading to many poor quality studies being conducted. The questions in this checklist are designed to identify the main features that should be present in a well designed study. There are few criteria that should, alone and unsupported, lead to rejection of a study. However, a study that fails to address or report on more than one or two of the questions addressed below should almost certainly be rejected.

For each question in this section you should use one of the following to indicate how well it has been addressed in the study:

- Well covered
- Adequately addressed
- Poorly addressed
- Not addressed (i.e. not mentioned, or indicates that this aspect of study design was ignored)
- Not reported (i.e. mentioned, but insufficient detail to allow assessment to be made)
- Not applicable.

2.1 THE STUDY ADDRESSES AN APPROPRIATE AND CLEARLY FOCUSED QUESTION

Unless a clear and well defined question is specified, it will be difficult to assess how well the study has met its objectives or how relevant it is to the question you are trying to answer on the basis of its conclusions.

2.2 THE DEFINITION OF MTBI IS CLEAR

Our case definition of MTBI is:

“MTBI is an acute brain injury resulting from mechanical energy to the head from external physical forces. Operational criteria for clinical identification include: (i) one or more of the following: confusion or disorientation, loss of consciousness for 30 minutes or less, post-traumatic amnesia for less than 24 hours, and/or other transient neurological abnormalities such as focal signs, seizure, and intracranial lesion not requiring surgery; (ii) Glasgow Coma Scale score of 13 – 15 after 30 minutes post-injury or later upon presentation for healthcare. These manifestations of MTBI must not be due to drugs, alcohol, medications, caused by other injuries or treatment for other injuries (e.g. systemic injuries, facial injuries or intubation), caused by other problems (e.g. psychological trauma, language barrier or coexisting medical conditions) or caused by penetrating craniocerebral injury.” (Carroll et al., J Rehabil Med, 2004, p. 115).

Persons with skull fractures included if they fit this case definition.

Additional from CDC: Abbreviated Injury Severity Scale (AIS) score of 2 for the head region; and the following ICD-9-CM codes.

ICD-9-CM First Four Digits =	ICD-9-CM Fifth Digit =
800.0, 800.5, 801.0, 801.5, 803.0, 803.5, 804.0, 804.5, 850.0, 850.1, 850.5 or 850.9	0, 1, 2, 6, 9, or Missing
854.0	1, 2, 6, 9, or Missing
959.0*	1

*The current inclusion of code 959.01 is provisional.

2.3 THE CASES AND CONTROLS ARE TAKEN FROM COMPARABLE POPULATIONS.

Study participants may be selected from the target population (all individuals to which the results of the study could be applied), the source population (a defined subset of the target population from which participants are selected), or from a pool of eligible subjects (a clearly defined and counted group selected from the source population. **If the study does not include clear definitions of the source population it should be rejected.**

2.4 THE SAME EXCLUSION CRITERIA ARE USED FOR BOTH CASES AND CONTROLS

All selection and exclusion criteria should be applied equally to cases and controls. Failure to do so may introduce a significant degree of bias into the results of the study.

2.5 WHAT PERCENTAGE OF EACH GROUP (CASES AND CONTROLS) PARTICIPATED IN THE STUDY?

Differences between the eligible population and the participants are important, as they may influence the validity of the study. A participation rate can be calculated by dividing the number of study participants by the number of eligible subjects. It is more useful if calculated separately for cases and controls. If the participation rate is low, or there is a large difference between the two groups, the study results may well be invalid due to differences between participants and non-participants. In these circumstances, the study should be downgraded, and rejected if the differences are very large.

2.6 COMPARISON IS MADE BETWEEN PARTICIPANTS AND NON-PARTICIPANTS TO ESTABLISH THEIR SIMILARITIES OR DIFFERENCES

Even if participation rates are comparable and acceptable, it is still possible that the participants selected to act as cases or controls may differ from other members of the source population in some significant way. A well conducted case-control study will look at samples of the non-participants among the source population to ensure that the participants are a truly representative sample.

2.7 CASES ARE CLEARLY DEFINED AND DIFFERENTIATED FROM CONTROLS

The method of selection of cases is of critical importance to the validity of the study. Investigators have to be certain that cases are truly cases, but must balance this with the need to ensure that the cases admitted into the study are representative of the eligible population. **The issues involved in case selection are complex, and should ideally be evaluated by someone with a good understanding of the design of case-control studies. If the study does not comment on how cases were selected, it is probably safest to reject it as a source of evidence.**

2.8 IT IS CLEARLY ESTABLISHED THAT CONTROLS ARE NON-CASES

Just as it is important to be sure that cases are true cases, it is important to be sure that controls do not have the outcome under investigation. Control subjects should be chosen so that information on exposure status can be obtained or assessed in a similar way to that used for the selection of cases. If the methods of control selection are not described, the study should be rejected. **If different methods of selection are used for cases and controls**

the study should be evaluated by someone with a good understanding of the design of case-control studies.

2.9 MEASURES WILL HAVE BEEN TAKEN TO PREVENT KNOWLEDGE OF PRIMARY EXPOSURE INFLUENCING CASE ASCERTAINMENT

If there is a possibility that case ascertainment can be influenced by knowledge of exposure status, assessment of any association is likely to be biased. A well conducted study should take this into account in the design of the study.

2.10 EXPOSURE STATUS IS MEASURED IN A RELIABLE WAY

Where exposure measures require any degree of subjectivity, some evidence should be provided that the measures used are reliable prior to their use in the study.

2.11 EXPOSURE STATUS IS MEASURED IN A VALID WAY

The primary exposure measures used should be clearly stated in the study. Where exposure measures require any degree of subjectivity, some evidence should be provided that the measures used have been validated prior to their use in the study.

2.12 THE MAIN POTENTIAL CONFOUNDERS ARE IDENTIFIED AND TAKEN INTO ACCOUNT IN THE DESIGN AND ANALYSIS

Confounding is the distortion of a link between exposure and outcome by another factor that is associated with both exposure and outcome. The possible presence of confounding factors is one of the principal reasons why observational studies are not more highly rated as a source of evidence. The report of the study should indicate which potential confounders have been considered, and how they have been assessed or allowed for in the analysis. Clinical judgement should be applied to consider whether all likely confounders have been considered. If the measures used to address confounding are considered inadequate, the study should be downgraded or rejected, depending on how serious the risk of confounding is considered to be. **A study that does not address the possibility of confounding should be rejected.**

2.13 CONFIDENCE INTERVALS ARE PROVIDED

Confidence limits are the preferred method for indicating the precision of statistical results, and can be used to differentiate between an inconclusive study and a study that shows no effect. Studies that report a single value with no assessment of precision should be treated with extreme caution.

SECTION 3 relates to the overall assessment of the paper. It starts by rating the methodological quality of the study, based on your responses in Section 2 and using the following coding system:

**++**	**All or most** of the criteria have been fulfilled.<br>Where they have not been fulfilled the conclusions of the study or review are thought *very unlikely* to alter.
**+**	**Some** of the criteria have been fulfilled.<br>Those criteria that have not been fulfilled or not adequately described are thought *unlikely* to alter the conclusions.
**-**	**Few or no** criteria fulfilled<br>The conclusions of the study are thought *likely or very likely* to alter.

The code allocated here, coupled with the study phase, will decide the **level of evidence** that this study provides. Phase 3 studies will be given the most weight.

3.4 PHASES OF STUDY

Phase 1: Studies are exploratory, hypothesis-generating studies characterised by descriptive explorations and demonstration of crude (unadjusted) associations.

Phase 2: Studies are also exploratory, but employ matching, stratification or multivariable analyses to identify independent prognostic factors, or employ multivariable models to predict outcomes.

Phase 3: Studies are confirmatory studies that test a priori hypotheses about one or more factors as independent predictors of outcomes, or groups of variables for clinical prediction. These studies include explicit control for confounding factors, or validate a clinical prediction rule.

The aim of the other questions in this section is to summarise your view of the quality of this study and its applicability to the patient group targeted.

Scottish Intercollegiate Guidelines Network,
Elliott House, 8-10 Hillside Crescent,
Edinburgh EH7 5EA
Web contact duncan.service@nhs.net
Last modified 21/04/11
© SIGN 2001-2011
