# Supplementary material for: Protocol for a systematic review of prognosis after mild traumatic brain injury: an update of the WHO Collaborating Centre Task Force findings
Source: Syst Rev. 2012 Feb 23;1:17. doi: 10.1186/2046-4053-1-17 (PMC3351713; doi:10.1186/2046-4053-1-17)
Supplement: Additional file 4 — Scottish Intercollegiate Guidelines Network (SIGN) considered judgment form. This file contains modified SIGN criteria and notes that were used to synthesize the evidence and make recommendations. [file 2046-4053-1-17-S4.PDF]

Considered judgement on quality of evidence

SIGN

Key question/area of interest:	Evidence table ref:

1. Volume of evidence

Comment here on any issues concerning the quantity of evidence available on this topic and its methodological quality.

2. Applicability

Comment here on the extent to which the evidence is directly applicable to the area of interest.

3. Generalisability

Comment here on how reasonable it is to generalise from the results of the studies used as evidence to the target population for this guideline.

4. Consistency

Comment here on the degree of consistency demonstrated by the available of evidence. Where there are conflicting results, indicate how the group formed a judgement as to the overall direction of the evidence

**5. Clinical impact**<br>*Comment here on the potential clinical impact that the intervention or factor in question might have – e.g. size of patient population; magnitude of effect; relative benefit over other management options; resource implications; balance of risk and benefit.*
**6. Other factors**<br>*Indicate here any other factors that you took into account when assessing the evidence base.*

7. **Evidence statement**<br>*Please summarise the development group's synthesis of the evidence relating to this key question, taking all the above factors into account, and indicate the evidence level which applies.*	**Evidence level high/moderate/ low**
8. **Recommendation**<br>*What recommendation(s) does the guideline development group draw from this evidence? Please indicate the grade of recommendation(s) and any dissenting opinion within the group.*	**Grade of recommendation**

SYNTHESISING EVIDENCE AND MAKING RECOMMENDATIONS

EVIDENCE TABLES

Once the assessment of the quality of evidence is complete, the next step is to extract the relevant data from each study rated as having a low or moderate risk of bias, and to compile a summary both of the individual studies, and the overall trend of the evidence.

SYSTEMATIC REVIEWS AND META ANALYSES

Systematic reviews are by definition summaries of results from a collection of other studies. Any attempt to summarise them further must be careful to ensure that all the issues addressed by the review are taken into account.

In the case of meta analyses where a very limited number of comparisons or measures of effect are used, the results can be presented in an evidence table along with any single studies that have been identified as addressing the same question.

Where multiple comparisons are made, with multiple measures of effect, or no meta analysis has been carried out by the reviewers, a separate summary table should be produced. For each review covered by the summary table, there should be an indication of the conclusions of the review and comment on its relevance to the key question being addressed. Where meta analyses have been carried out on a range of comparisons and/or outcomes the results of these calculations should be tabulated in the summary. **Guideline developers should still refer to the full text of the systematic review when making their judgements on the evidence.**

EVIDENCE OF DIAGNOSTIC ACCURACY

Where there are a number of studies addressing the accuracy of diagnostic tests, an evidence table summarising key aspects of the studies is produced. These tables are produced in a standard format that includes the following columns in addition to identification of the study and its methodological quality:

- Number of patients included in the study
- Prevalence of the condition being tested for in the study population
- Characteristics of the study population
- The type of test being evaluated
- Reference standard with which the new test was compared.
- Sensitivity of the test
- Specificity of the test
- Positive predictive value of the test
- Negative predictive value of the test
- Likelihood ratios for the test
- Source of funding for the study
- Specific issues raised by the study that are relevant to the question being addressed.

All measures of accuracy reported in each study should be included, though only rarely will all those listed appear in every study. To make cross-study comparisons possible, measures may be calculated from the data presented in the studies and included in the evidence table. Likelihood ratios will normally be used for this purpose. Where results are calculated rather than taken from the original papers, this should be indicated in the evidence table.

OTHER TYPES OF STUDY

All studies other than systematic reviews or diagnostic studies will be summarised in a general evidence table. The standard format for these tables includes columns covering the following data in addition to identification of the study, its type, and its methodological quality:

- Number of patients included in the study
- Characteristics of the patient population
- Intervention, risk factor, etc. being investigated in the study
- Comparisons made in the study
- Length of follow-up
- Outcome measures used
- Effect size, including statistical measures such a p values or confidence intervals
- Source of funding
- Specific issues raised by the study that are relevant to the question being addressed

Given that the process of carrying out a methodological appraisal inevitably involves a certain amount of subjective judgement, guideline development groups are asked to ensure that each study is independently evaluated by *at least two* individuals and consensus reached on the rating before it is included in any evidence table.

CONSIDERED JUDGEMENT

Once the evidence has been compiled into an evidence table, the development group need to decide what recommendations can be made on the basis of this evidence. This is perhaps the most difficult part of the whole process, and requires the exercise of judgement based on experience as well as knowledge of the evidence and the methods used to generate it. Although it is not practical to lay out 'rules' for the exercise of judgement in this way, some of the most important issues that should be considered by guideline development groups are discussed below. The principal headings under which these are considered are:

- Quantity, quality, and consistency of evidence
- Generalisability of study findings
- Directness
- Clinical impact

This list is not exhaustive, nor does it seek to explain the complete background to the factors identified. Guideline development groups are encouraged to use sensitivity analysis of their evidence tables to see whether cutting out particular quality levels or types of study makes a material difference to the results. They are also encouraged to take into account other factors that they deem appropriate, and should keep a record of what factors have been considered, analyses undertaken, and decisions reached in respect of each recommendation they make. An example proforma for this is included in the companion document.

Increasing the role of subjective judgement in forming guideline recommendations necessarily increases also the risk of re-introducing bias into the process. However, it must be emphasised that this is not the considered judgement of an individual, but of a carefully composed multidisciplinary group. An additional safeguard is the requirement for the guideline development group to present clearly the evidence on which the recommendation is based, making the link between evidence and recommendation explicit and explaining how and why they have exercised their considered judgement in the interpretation of that evidence. Wherever possible, guidelines should also present clear and succinct information on, e.g. absolute and relative risk reduction, and numbers needed to treat to achieve a defined benefit in order to enable local decision-makers, individual clinicians and patients to understand the basis of the recommendation and to make a judgement as to whether it applies in their individual circumstances.

CRITICAL OUTCOMES

When the evidence collected to answer a particular question is first reviewed, it is quite common to find that a wide range of outcome measures. Some of these are likely to be direct measures of effect such as prolonged survival time, improved lung function, etc. Others may be surrogate measures such as reduced drug use or improved quality of life. Guideline developers have to consider the range of outcome measures used, and decide which of these will be critical to the decisions they make. In most cases the critical outcomes will be direct measures of effect, but in some circumstances less direct measures may be given higher importance. Patients may give higher importance to improvements in quality of life than to marginal improvements in survival time, for example. For this reason it is important that all members of the guideline group participate in the process of agreeing on critical outcomes.

To establish the critical outcomes, all outcomes used in the evidence tables should be listed. (Note that it is important that any harms associated with a proposed intervention are included in the list of outcomes.) The group must then reach consensus on the relative importance of each measure on a scale from one to nine, with one representing least importance and nine the highest. Those outcomes with an agreed importance of seven or higher may be regarded as key outcomes.

Once the critical outcomes have been agreed, subsequent discussions should focus on these outcomes. The final level of evidence will be based on **the lowest level of evidence applicable to a key outcome**. This conservative approach to evaluation of the evidence will reduce the risk of the benefits of an intervention being overstated.

QUANTITY, QUALITY AND CONSISTENCY

The aim of SIGN guidelines is to provide national guidance about effective clinical practice for the management of patients within the NHS in Scotland. It is important to ensure that recommendations are supported by an adequate evidence base. Guideline development groups should consider the number of studies (including the overall number of patients studied) and should be cautious about making strong recommendations based upon a small number of studies, a set of studies with small sample sizes, or poor quality studies.

The quality of the methodology used in studies is addressed through the use of the checklists during the quality rating step of the grading process. These quality assessments focus on the risk of bias and do not consider other issues such as the statistical power of studies. Small, under powered studies that are otherwise sound may be included in the evidence base if their findings generally point in the same direction – but at least some of the studies cited as evidence must be large enough to detect the size and direction of any effect.

In evaluating the evidence base and deriving recommendations, guideline development groups need to look at the results across all the studies identified to explore whether the findings appear to be reasonably consistent across the range of study populations and study designs. If a statistical analysis of heterogeneity has been carried out and finds little evidence of inconsistency, decisions are comparatively straightforward. If, however, the calculation demonstrates a high degree of heterogeneity or no such calculation has been done, guideline developers need to explore potential reasons for the variation and consider the implications for the recommendations which that evidence base can support.

Factors which might introduce heterogeneity into study findings include:

i. *Type of study design*
   There is considerable evidence that non-randomised studies of the effectiveness of health care interventions tend to have larger estimates of effect as a result of the greater bias in such studies. Guideline development groups should consider whether the findings are consistent across different study designs; if they are not, greater weight should be given to the findings from study designs higher up in the hierarchy. However, the importance of non-randomised studies in confirming or questioning results from randomised trials should be borne in mind.

ii. *Quality of studies*
   There is considerable evidence that aspects of the conduct of studies can lead to biased findings. The quality criteria used to appraise studies as part of the SIGN guideline development process reflect aspects of the conduct of studies that may introduce bias. In the case of observational study types, they also try to assess the risk of confounding inherent in the study design, and the likelihood of the results being attributable to chance. Guideline development groups should consider whether the findings are consistent across studies of variable quality within the same

broad study design category. If they are not, greater weight should be given to findings from well conducted studies.

iii. *Variations in population*
Evidence should be reviewed to assess whether differences in study population could explain some or all of the variation. Biological (age, gender mix, disease status) or socio-economic (social group, employed/unemployed, etc.) factors can all have an effect on outcome.

GENERALISABILITY

The generalisability of the evidence should also be considered when weighing up conflicting results. Whilst the conclusions drawn from a well-conducted RCT may be valid in themselves – i.e. the *efficacy* of the intervention in question may have been proven in the context of that trial; unless this result is generalisable beyond the trial population and setting, the *effectiveness* of the intervention has not been established. In this situation, care must be taken not to automatically give more credence to evidence from RCTs vs. conflicting evidence from observational studies without careful consideration of the appropriateness of the trial setting and the entry conditions which were applied to the trial population.

Of course, the converse of this also applies: if evidence from RCTs is *confirmed* by observational studies in clinical practice, then the recommendation will be all the more robust. The ideal would be to establish efficacy through the use of RCTs, and subsequently establish effectiveness by carrying out trials that are randomised but have very broad entry criteria (i.e. including all patients who in the "real world" might be prescribed the drug or intervention in question). As yet, however, evidence on effectiveness from this kind of trial may not be widely available.

DIRECTNESS (APPLICABILITY)

The findings of the most rigorously conducted studies may not be directly relevant to the development of guideline recommendations if they are evaluating an intervention or factor which is not available or applicable to the area of interest. In such cases recommendations should be made on the basis of the best evidence that is applicable in the appropriate context. Reference should be made to any other evidence that has been identified and evaluated, and an explanation provided as to why the results have not been accepted for the guideline.

In some cases it may be necessary to base recommendations on extrapolated data from studies of other populations. Guideline development groups need to consider carefully the application of study findings to certain patients and settings, and be aware that they represent a weakening of the evidence base and consequent downgrading of any associated recommendations.

One commonly used method of assessing applicability of evidence is to compare the characteristics of the study population with those of the population we wish to apply the intervention or factor to. However this is probably an over simplistic approach and can lead to inappropriate conclusions unless other factors, such as the study setting, are also taken into account.

An alternative approach is to consider which underlying biological or social factors might limit the applicability of study findings and make judgements about whether there are sufficient differences in these factors to justify *not* applying the evidence to a certain population. Effect modifying factors which might reduce the direct application of study findings include

i. *Patient factors*
Guideline development groups need to consider whether there are biologically plausible factors which might modify the relative importance of different prognostic factors, sensitivity and specificity of diagnostic tests, or effects of interventions and that differ between the populations in identified studies and a certain population. Examples of such factors include baseline risk with reference studies in primary and secondary care, or the influence of gender, age, or ethnicity.

ii. *Provider factors*
Guideline development groups need to consider whether there are provider or organisational factors which might modify the sensitivity or specificity of diagnostic tests of the effects of interventions, e.g. the availability of experienced staff to undertake the procedure or to interpret the results.

iii. *Cultural factors*

Guideline development groups also need to consider whether there are cultural factors that might modify the relative importance of different prognostic factors, the sensitivity and specificity of diagnostic tests, or the effects of interventions and which differ between the populations in identified studies and a certain population. Examples of such factors include attitudes to health promotion, or attitudes to sexuality.

In the case of studies of diagnostic tests there are a number of additional criteria that need to be taken into account to ensure that the findings are relevant to the population targeted by a guideline. These include:

i. *Spectrum of disease*

Does the study cover a range of disease states? This is particularly important for long term diseases such as cancer, where patients may have anything from early stage pre-metastatic disease to advanced cancer. The tests studied should demonstrate a high degree of specificity and sensitivity across a range of disease states. The range must be clearly indicated, as the results cannot be generalised to other disease states.

ii. *Study setting*

This is influenced by the patient group studied. In primary care, there is likely to be a mix of diseased and undiseased subjects; in tertiary care, all subjects can be expected to have advanced disease. Knowledge of the social background of patients or the existence of an epidemic of a disease at the time of the study may predispose investigators to identify as positive results that would at other times be seen as marginal or negative.

iii. *Duration of illness*

How easy is the illness to diagnose? How long does it need to be present before the test will find consistent results? The population to be studied should be selected to take this into account

CLINICAL IMPACT

The final issue for guideline development groups to consider in deriving recommendations from the validated evidence base that they have assembled is whether the potential benefit from application of the intervention, test etc. in question is sufficiently great to justify a recommendation that it should be used in practice. This will depend on a large number of factors, including the size of the patient population concerned, the magnitude of the effect compared with no intervention or other management options, the duration of therapy required to achieve the effect, and the balance of risk and benefit.

If no economic studies have been included in the evidence base, consideration should be given as to whether any recommendation based on the evidence will have resource implications for the area of interest. The questions that should be considered include:

1. Does the proposed course of action involve the use of resources currently available to the area of interest?

2. If not, are the resources likely to be available tothe area of interest?

3. If the required resources are unlikely to be widely available, is there evidence to support an alternative course of action?

4. Is implementation of the recommendation likely to require new resources, or the reallocation of existing resources?

5. If so, how significant is the demand for additional resources likely to be? Would a full-scale economic analysis of the options be justifiable?

(Note that “resources” covers a wide range of items other than financial resources – trained staff, clinic time, specialised diagnostic or treatment facilities, etc.)

This stage should also include consideration of patient concerns and wishes in relation to the treatment that is offered. Evidence that patients are willing to comply with the treatment provided should strengthen a recommendation. Alternatively, evidence that patients are unable or unwilling to follow prescribed treatments should weaken a recommendation and link it to the need for research into the reasons underlying patient concerns, and how they can be addressed.

Scottish Intercollegiate Guidelines Network,

© SIGN 2001-2011
